# Estimation of the Postmortem Duration of Mouse Tissue by Electron Spin Resonance Spectroscopy

**DOI:** 10.1155/2011/973172

**Published:** 2011-06-27

**Authors:** Shinobu Ito, Tomohisa Mori, Hideko Kanazawa, Toshiko Sawaguchi

**Affiliations:** ^1^I.T.O. Provitamin Research Center, 1-6-7-3F Nakamachi, Musashino, Tokyo, Japan; ^2^Faculty of Pharmacy, Keio University, 1-5-30 Shibakoen, Minato-ku, Tokyo 105-8512, Japan; ^3^Department of Pharmacology and Experimental Neuroscience, College of Medicine, University of Nebraska at Omaha, Omaha, NE 68182, USA; ^4^Department of Occupational Therapy, Faculty of Regional Health Therapy, Teikyo Heisei University, 4-1 Uruido-minami, Ichihara, Chiba, Japan

## Abstract

Electron spin resonance (ESR) method is a simple method for detecting various free radicals simultaneously and directly. However, ESR spin trap method is unsuited to analyze weak ESR signals in organs because of water-induced dielectric loss (WIDL). To minimize WIDL occurring in biotissues and to improve detection sensitivity to free radicals in tissues, ESR cuvette was modified and used with 5,5-dimethtyl-1-pyrroline N-oxide (DMPO). The tissue samples were mouse brain, hart, lung, liver, kidney, pancreas, muscle, skin, and whole blood, where various ESR spin adduct signals including DMPO-ascorbyl radical (AsA^*∗*^), DMPO-superoxide anion radical (OOH), and DMPO-hydrogen radical (H) signal were detected. Postmortem changes in DMPO-AsA^*∗*^ and DMPO-OOH were observed in various tissues of mouse. The signal peak of spin adduct was monitored until the 205th day postmortem. DMPO-AsA^*∗*^ in liver (*y* = 113.8–40.7 log (day), *R*1 = −0.779, *R*2 = 0.6, *P* < .001) was found to linearly decrease with the logarithm of postmortem duration days. Therefore, DMPO-AsA^*∗*^ signal may be suitable for detecting an oxidation stress tracer from tissue in comparison with other spin adduct signal on ESR spin trap method.

## 1. Introduction

Electron spin resonance (ESR) or electron paramagnetic resonance (EPR) is now widely used to analyze free radical species in living body and materials. Possibility of application of ESR is studied in a forensic science area. It can be potentially used for estimating postmortem duration in the cause of death. Pashinian and Proshut [1], who suggested the potential of using ESR in forensic medicine, attempted to determine the time of the occurrence of mechanical trauma by measuring the ESR signals of bone marrow. Several studies have analyzed blood by ESR, because blood contains iron-containing proteins such as hemoglobin. Uzeneva [2], for example, studied on the ESR signals of posttraumatic blood. Mil' et al. [3] reported that the ESR signal intensity of blood of patients exposed to radiation at the Chernobyl nuclear accident is higher than that of healthy people. Nakamura et al. [4] reported on ESR signals induced by ionizing radiation in teeth. Quarino and kobilinsky. [5] used ESR to detect human hemoglobin from bloodstains. Türkes et al. [6] analyzed blood stored under blood bank conditions using ESR. They reported that the intensity of ESR signals from methemoglobin, nonheme irons, and organic radicals in dried human blood increase with time. Fujita et al. [7] showed that (1) ESR signals from bloodstains are effective in estimating the age of human and (2) ESR signals regularly change over time within the period of 432 days. In these ESR studies, measurements were performed at low temperature (140°K) for detecting the ESR signal of protein-bonded ions. As described above, ESR is now widely used to analyze living body and material in forensic medicine, and it can be potentially used to estimate the age of human from bloodstains. In those cases, ESR measurements were performed at room temperature unless otherwise mentioned. However, with the exception of blood, few studies have examined the postmortem changes in ESR signals found in organs and tissues. In this study, we investigated the origins of ESR signals in postmortem tissues and the time courses of changes in the signals. 

ESR spin trapping and probing is a method that has recently attracted attention and is used to analyze the free radicals of tissues. ESR spin trapping method is performed by a conventional X-band ESR analysis system [8, 9], which detects individual radical types as spin adducts and identify and quantify reactive oxygen species (ROS) types based on the signal patterns. ESR spin probing method has recently been applied to three-dimensional ESR imaging for living body [10–14], but the method has to analyze relatively weak signals from living body [15, 16]. Ascorbic acid (AsA) is a superior scavenger; it reacts with hydroxyl radicals strongly, the rate of reaction is 7.0 × 10^9^–1.1 × 10^10^ M^−1^S^−1^ [17], and Ascorbyl radical (AsA*) is generated after the reaction. The detection of ESR signals of AsA* is straightforword, because the spin trap adduct signal of AsA* is simple. AsA* has a possibility as an important indicator for oxidation stress in tissue. Previous study of AsA* spin adduct signal was limited to tissues having strong oxidative stresses or AsA administration mouse having a high AsA* level. A doublet peak spectrum was found to obtain following AsA injection in mouse, and the signals were confirmed in different ways due to AsA* [18]. It was reported that (1) tissue constantly suffers from the oxidation of AsA and iron proteins [19] and (2) the oxidation reaction could proceed by the reaction of AsA by these recycle Fenton reactions [20]. AsA* was detected in those tissue suffering from oxidative stress. DMPO-(5,5-dimethtyl-1-pyrroline N-oxide) AsA* was detected in oxidative stress mouse skin induced by X-ray irradiation [21]. A method to detect DMPO-AsA* signal with a high sensitivity from a brain was reported recently by Masumizu et al. [22]. Since ESR signals of the tissues are extremely weak, the detection of signals has been difficult by conventional methods due to water-induced dielectric loss (WIDL). However, the detection of DMPO-AsA* from a normal organ without oxidation stress was also difficult. To minimize WIDL in biotissues, we attempted to detect tissue free radicals by modifying ESR cuvettes and using DMPO. As normal tissue samples, brain, hart, lung, liver, kidney, pancreas, muscle, skin, and whole blood of mice were used. From these tissues, various ESR spin adduct signals including DMPO-AsA*, DMPO-superoxide anion radical (OOH), and DMPO-hydrogen radical (H) signal were detected. The postmortem changes in AsA spin adduct and other signals were monitored up to 205 days. Possible application of AsA* adduct signal as a natural oxidation stress indicator was also investigated through these experiments.

## 2. Materials and Methods 

### 2.1. Generation of Standard Free Radicals and the Measurement of ESR Signal

In accordance with the methods of Masumizu et al. [22], multiple standard free radicals were generated by following the radical generation system, and the *g*-value and hfcc of each spin adduct were obtained by ESR-spin trapping method. A spin trapping agent (10–50 *μ*L) and a reaction liquid of the following free radical generation system (10–50 *μ*L) were placed on a high purity quartz cuvette, which was covered with a cover glass (0.15 mm in thickness), and spin adduct signals were measured by an ESR. Cover glass was bonded to the cuvette with the surface tension of spin trapping agent. Spin trapping agents used in this study were DMPO (100 w/w%, liquid), 5-(dipropoxy phosphoryl)-5-methyl-1-pyrroline N-oxide (DPPMPO) (50–500 mM, dimethyl sulfoxide solution). The signal ratio was obtained for each measurement using the signal of MnO, an internal standard substance, as a standard.

### 2.2. Standard Free Radical Adduct Signal

Standard ESR signals of various free radicals were provided by the following systems. 

#### 2.2.1. Superoxide Anion Radical Generation System

Superoxide was generated with a hypoxanthin-xanthine oxidase reaction system. Superoxide dismutase (SOD) solution (30 *μ*L) (0.1 phosphate buffer/saline, pH 7.8, 200 U/mL) was added to it, and an appeared peak is assigned to DMPO-OOH (superoxide radical) or DPPMPO-OOH.

#### 2.2.2. Hydroxyl Radical Generation System

Hydroxyl radicals were generated from the reaction of 10 mmol/L FeSO_4_ and 20 mmol/L H_2_O_2_ (Fenton reaction). Hydroxyl radical were confirmed by adding 30 *μ*L AsA solution (0.1 mol/L AsA), and tan appeared peak is assigned to DMPO-OH (hydroxyl radical) or DPPMPO-OH.

#### 2.2.3. AsA* Generation System

AsA* was generated by reacting hemoglobin (0.1 w/w%) and AsA (1 mmol/L). AsA* was also generated by adding 10 *μ*L L-ascorbic acid solution (10 mmol/L) to the 10 *μ*L hydroxyl radical generation system described above.

#### 2.2.4. Hydrogen Radical

Hydrogen radical was generated by hematoporphyrin (1 w/w%) with UV irradiation at 365 nm (the intensity: 5 mW/cm^2^) (Ushio Optical Modulex, SX-UI 500MQQ)(Ushio, Tokyo, Japan). Hydrogen radical is also generated by electrolyzing 0.01 w/w% NaCl solution with TI-8000 (Nihon Trim, Osaka, Japan). For confirming the generation of hydrogen radicals, DBNBS was added to the hydrogen radical solution and the color of the solution was observed to be orange (P2002-350420A). The *g*-value of free radical signal obtained and identifyied and the signal was identified by the calculation of both frequency and magnet field of the ESR signal. For correcting internal cavity for quantitative analysis, manganese oxide (MnO) was used as the internal standard of ESR cavity. DMPO signals were recorded between 3rd and 4th MnO signals. The relative intensity of radicals was calculated by comparison with the 3rd MnO signal intensity. The *g*-value and the distance (mT) between the peaks for hfcc were measured by software coming with ESR device. ESR equipment and its condition used in this study were followings. The measurements of *g*-value and hfcc were calculated by analysis software (A-System vl.40 ISAJ, FA-manager vl.20, JES, Tokyo, Japan) accompanying with ESR spectrometer. Numerical value was measured more than three times, and the numerical maximum dispersion range is shown in ± number.

### 2.3. Equipment

Electron spin resonance (ESR) spectrometer (JEOL, JES-FA200 spectrometer, Tokyo). ESR spectrometry conditions used to estimate each radical with spin-trapping reagent were as follows: microwave frequency: 9414.499 ± 5.000 MHz, microwave power: 4.00 mW, field center: 335.32 ± 0.5 mT, sweep width: ±5.00 mT, modulation frequency: 100.00 kHz, modulation width +/−: 0.1 mT, sweep time: 0.5–5 min, amplitude: 1.500–2.500, and time constant: 0.03–0.5 s, at room temperature. ESR universal cavity (JEOL, ES-UCX2 : TE011 mode cavity) with an X-band microwave unit (8.750–9.650 GHz). ESR standard marker: manganese oxide (MnO) powder (JEOL DATUM, MO7-FB-4) aqueous sample cell (JEOL, ES-LC12), sample volume: 20–100 *μ*L. A tissue-type quartz cell (Labotec, Tokyo) with home-made cover glass (Size: 40 × 5 × 0.5 mm in thickness).

### 2.4. ESR Signal Measurement in Animal Tissue

#### 2.4.1. Animals

For postmortem change experiments, male ddY mice (Nihon SLC, Shizuoka, Japan) weighing 20.1–25.7 g (6 to 8 weeks old) were used. The animals were housed at a room temperature of 20.2–25.3°C under a 12-h light-dark cycle (lights on at 7:00 a.m.). Food and water were available ad libitum. All of the following procedures were conducted in accordance with the guiding principles for the care and use of laboratory animals promulgated by the Japanese Pharmacological Society and with the guidelines for animal care in our laboratories, as approved by the Tokyo Women's Medical University Committee on animal care and use. Food was withdrawn 24 h before experiments. 

#### 2.4.2. Removal of Tissue and ESR Analysis

Mice were sacrificed by dislocating their cervical spine. The tissues were immediately removed and placed on an ice-cold plate after being rinsed with ice-cold buffer (0.1 mol/L phosphate buffer/saline, pH 7.8). The tissues were sliced into 0.2–0.3 mm in thickness using a microtome (KN3150465) (Kenis, Osaka, Japan). Slice weight was measured for normalizing ESR signal of each radical. Brain tissues were removed from the cerebral hemisphere. Hart tissues were removed from the lower tip of the atrium. Tissues of lung, liver, kidney, and pancreas were collected. Muscle tissues were removed from the thigh muscle of right legs. Skin tissues were removed from the tip of ears. Whole blood was sampled from the heart. DMPO (10–50 *μ*L) was added to the tissue samples (10–50 mg) or the blood (10–50 *μ*L) immediately after being weighed, and at precisely five minutes after remove, ESR signals were measured. To identify obtained peaks, the signals measured were analyzed by specialized analysis software, installed in the ESR device, for determining the *g*-value and hfcc calculated from the distance between peaks. After the adduct signals of superoxide were confirmed, the decaying of the peak was monitored by adding SOD solution to cuvettes containing samples. 

### 2.5. Postmortem Change

Mice were sacrificed by dislocating their cervical spine. Their tissues were collected, and their ESR spin adduct signals were detected by the procedures described above. For observing postmortem change of ESR signal, the sliced tissues were stored at 4°C and for 3–125, 163, 205 days after being sealed with polyvinylidene chloride film to prevent water evaporation for creating fixed decomposition conditions. The sliced mouse tissues were ESR-analyzed on the 3, 15, 30, 60, 125, 163, and 205 days postmortem by the procedures described above.

### 2.6. Chemicals

The chemicals used in the present study were 5,5-dimethyl-1-pyrroline-N-oxide (DMPO) (Labotec, Tokyo, Japan), 5-(dipropoxy phosphoryl)-5-methyl-1-pyrroline N-oxide (DPPMPO), (Dojin chemicals, Kumamoto, Japan), xanthine oxidase (MP Biomedicals, Ohio, USA), hypoxanthine (Wako Pure Chemical Industries, Osaka, Japan), methanol, (USP grade), dimethyl sulfoxide, sequencing (DMSO) (Pierce Biotechnology, Ill, USA), hydrogen peroxide (Wako Pure Chemical,) ferrous sulfate, (USP grade), superoxide dismutase, from bovine erythrocytes (Cu/Zn Type) (Wako Pure Chemical), L(+)- ascorbic acid (Wako Pure Chemical), and 3,5-dibromo-4-nitrosobenzenesulfonic acid sodium salt (MP Biomedicals, Inc., Ohio, USA). All other chemicals were of analytical grade.

### 2.7. Data Analysis

Standard ESR signals were recorded on the ESR computer system, the position and height of peaks were recorded together with the height of the internal standard. *g*-value and hfcc were calculated automatically after measurements with ESR computer software. The ESR signals of samples were identified by determining the *g*-value and hfcc of measurable peaks and compared with the peak values of standard radicals. 

### 2.8. Statistical Analysis

Data are expressed as the mean with S.E.M. One-way ANOVA followed by Dunnett's multiple comparison test was used for evaluating the significance of difference. A *P* less than <.05 was considered significant. In the analysis of an ESR signal of postmortem change, regression line, correlation coefficient (*R1*), contribution rate (*R2*), and the significant difference calibration were calculated by a method of Pearson. A correlation coefficient (*R1*) ≧ 0.7 and a contribution rate ≧ 0.5 were considered significant. 

## 3. Results

**Figure 1:**
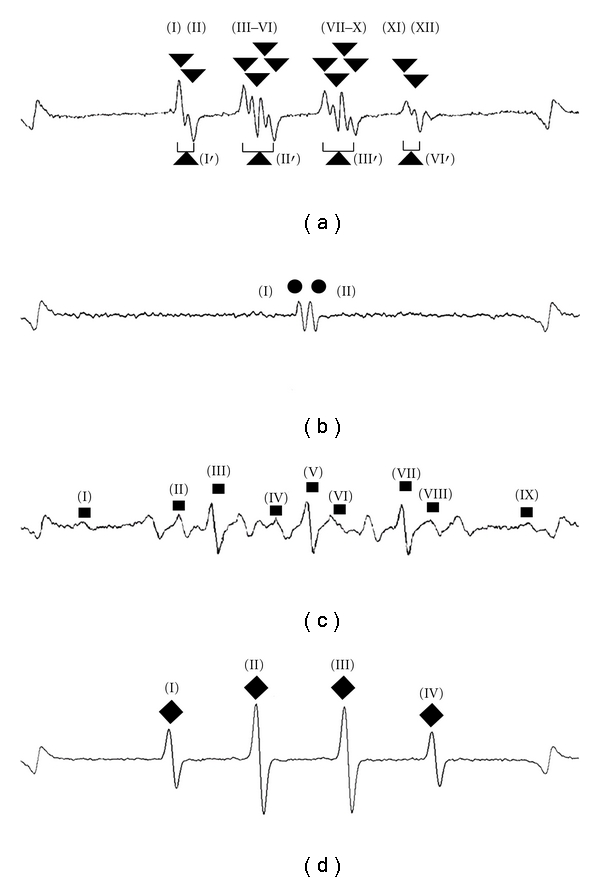
Standard ESR signals of DMPO- and DPPMPO-adducts.

Signals A, B, C, and D shown in Figure 1 were originated from DMPO-OOH (superoxide adduct signal), DMPO-AsA* (AsA radical adduct signal), DMPO-H (hydrogen radical adduct signal), and DMPO-OH (hydroxyl radical adduct signal), respectively. The hfcc of DMPO-OOH, DMPO-AsA*, and DMPO-H was in agreement to three digits after decimal point within the max range of ±0.0009, and this value was almost the same as the previous study [20]. With regard to DMPO-OOH, when peaks decayed, adjacent peaks were merged, making it difficult to distinguish their *g*-values, because aH*γ* values (0.132) was quite small. Therefore, the *g*-values of combined adjacent peaks were determined by the standard peaks in advance, and the *g*-values and the distance between the combined peaks were used for identification. The following *g*-values were determined from the standard waveform and used to identify the peaks detected in the organs when peaks decayed in tissue. For example, in Figure 1(a), (I′) was the center point *g*-value between the *g*-value of the highest point of peak (I) and the *g*-value of the lowest point of peak (II) (2.0173). (II′) was the center point the *g*-value between the *g*-value of the highest point of peak (III) and the *g*-value of the lowest point of peak (VI) (2.0096). Further tests for identification showed that the peak of DMPO-OH was decayed with the addition of AsA solution, while the peak of DMPO-OOH was decayed with the addition of SOD solution. The existence of hydrogen radicals was confirmed by observing orange color in the DDTMK-added hydrogen-radical solution. These results confirmed that the peaks of the spin adducts artificially generated were adequate as standard peaks. The *g*-value and hfcc of DMPO-AsA* signal were 2.0045 and 0.187, respectively. These values were almost the same as the previous study [22]. The tissue samples were the brain, hart, lung, liver, kidney, pancreas, muscle, and skin of ddy mice. Various ESR spin adduct signals including AsA signal were detected in these samples. These results showed that DMPO-AsA*, DMPO-OOH, and DMPO-H were detectable and identifiable in the mouse tissue. 

**Figure 2:**
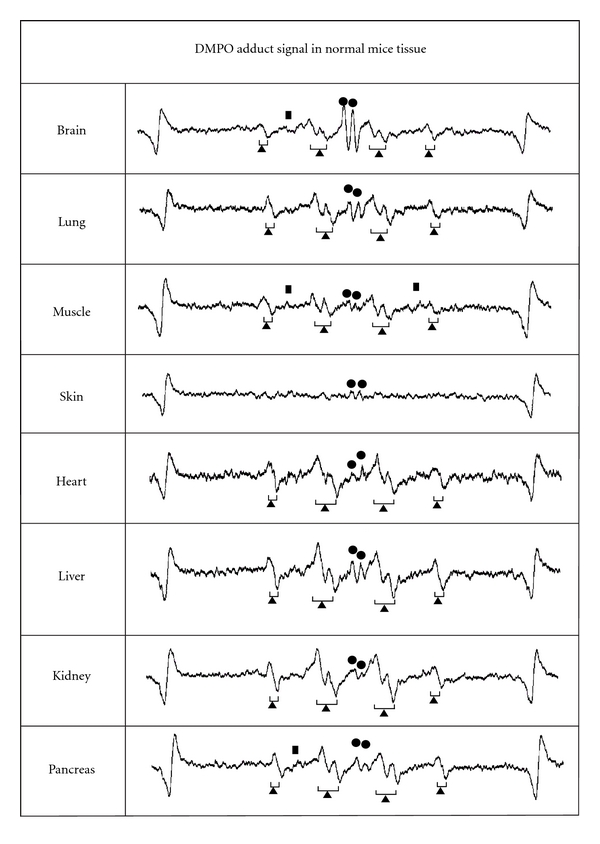
The representative examples of DMPO adduct signals from various mice tissues. The marks (●,▲, and ■) show the peaks that were the same peaks having the *g*-value of standard signal.

**Figure 3:**
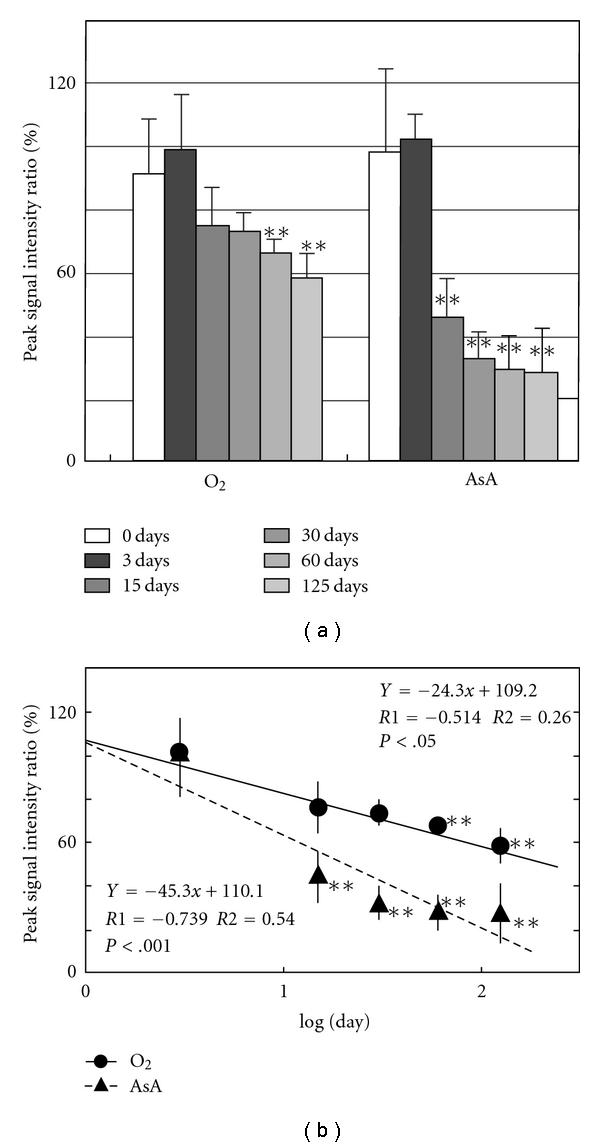
(a) Postmortem DMPO-adduct signal intensity in mouse brain. The columns and the lines show the postmortem signal intensity ratios of brain and S.E.M. of the means (*n *= 8). The *y*-axis shows the percentage of peak signal intensity ratio, which was calculated by assuming that the intensity of MnO (500 nmol/L) was 100%. *P < .05, **P < .01, versus 0 days. (b) Time course of postmortem DMPO-adduct signal intensity ratio in mice brain. The *Y*-axis shows the percentage of peak signal intensity, which was calculated by assuming that the intensity at 3 day postmortem was 100%. *X*-axis is expressed in Log(day). *P < .05, **P < .01, versus 3 days. The regression lines, correlation coefficient (*R1*), the square of *R1* (*R2*) and the probability of error (*P*-value) were calculated.

**Figure 4:**
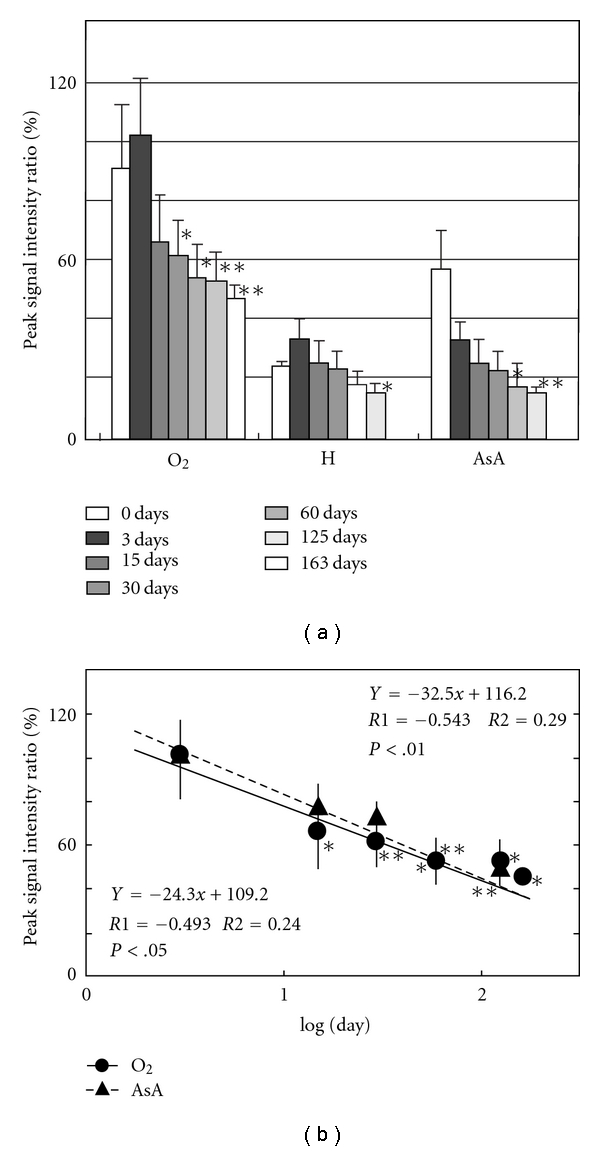
(a) Postmortem DMPO-adduct signal intensity in mouse lung. The columns and the lines show the postmortem signal intensity ratios of brain and S.E.M. of the means (*n* = 8). The *y*-axis shows the percentage of peak signal intensity ratio, which was calculated by assuming that the intensity of MnO (500 nmol/L) was 100%. *P < .05, **P < .01, versus 0 days. (b) Time course of postmortem DMPO-adduct signal intensity ratio in mouse lung. The *y*-axis shows the percentage of peak signal intensity, which was calculated by assuming that the intensity at 3 day postmortem was 100%. *X*-axis is expressed in log(day). *P < .05, **P < .01, versus 3 days. The regression lines, correlation coefficient (*R1*), contribution rate (*R2*), and the probability of error (*P*-value) were calculated.

**Figure 5:**
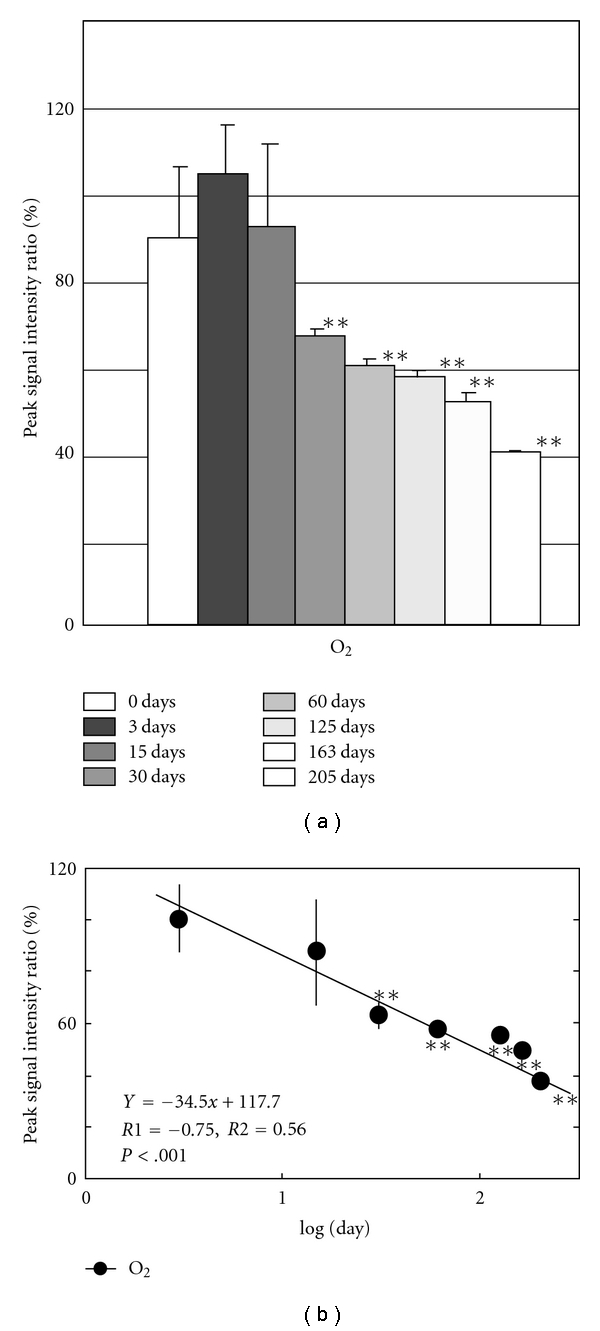
(a) Postmortem DMPO-adduct signal intensity in mouse heart. The columns and the lines show the postmortem signal intensity ratios of brain and S.E.M. of the means (*n* = 8). The *y*-axis shows the percentage of peak signal intensity ratio, which was calculated by assuming that the intensity of MnO (500 nmol/L) was 100%. *P < .05, **P < .01, versus 0 days. (b) Time course of postmortem DMPO-adduct signal intensity ratio in mouse heart. The *y*-axis shows the percentage of peak signal intensity, which was calculated by assuming that the intensity at 3 day postmortem was 100%. *X*-axis is expressed in Log(day). *P < .05, **P < .01, versus 3 days. The regression lines, correlation coefficient (*R1*), contribution rate (*R2*), and the probability of error (*P*-value) were calculated.

**Figure 6:**
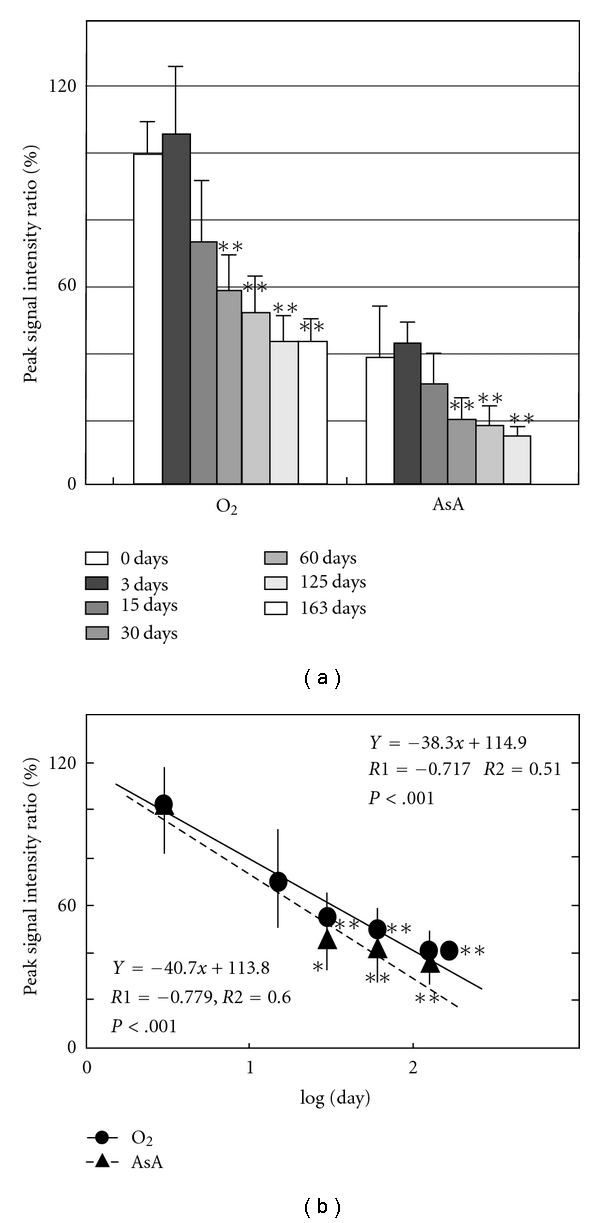
(a) Postmortem DMPO-adduct signal intensity in mouse liver. The columns and the lines show the postmortem signal intensity ratio of brain and S.E.M. of the means (*n* = 8). The *y*-axis shows the percentage of peak signal intensity ratio, which was calculated by assuming that the intensity of MnO (500 nmol/L) was 100%. *P < .05, **P < .01, versus 0 days. (b)Time course of postmortem DMPO-adduct signal intensity ratio in mouse liver. The y-axis shows the percentage of peak signal intensity, which was calculated by assuming that the intensity at 3 day postmortem was 100%. *X*-axis is expressed in Log(day). *P < .05, **P < .01, versus 3 days. The regression lines, correlation coefficient (*R1*), contribution rate (*R2*) and the probability of error (*P*-value) were calculated.

Figure 2 shows ESR signals of DMPO adduct of normal mice from brain, lung, muscle from femoral, skin from ear, heart, liver, kidney, and pancreas. The marks (●, ▲, and ■) were added to the peak that accords with the g-value of standard signal in Figures 1 and 2. Measurement of the standard waveform and hyperfine structure constant of free radicals was performed. Figure 1 shows the spectrum of DMPO standard adduct signals of various free radicals that were artificially generated. Postmortem changes in DMPO-AsA∗ and DMPO-OOH signals were observed at 4 °C in various tissues of the mouse.  Figure 3(a) shows the change of signal from brain; Figure 4(a), lung; Figure 5(a), heart; Figure 6(a), liver. The brain was measured up to 125 days before tissue destruction, the lungs and the liver were able to be measured up to 163 days, and the heart, a strong tissue, was measured up to 205 days. The signal peaks of the spin adducts identified were monitored at the 125, 163, and 205th day after postmortem. The increase of signal intensity ratio was observed from 0 to 3 days posthumously but no significant difference.

All peak intensity ratios decreased postmortem. The decrease rate was found to be straight on the logarithmical scale of postmortem period. The regression analysis of the signal intensity ratio and the postmortem period was performed for each tissue. The results of regression analysis of brain were shown in Figure 3(b); lung, Figure 4(b); heart, Figure 5(b); liver, Figure 6(b). Furthermore, correlation coefficient (*R1*), the square of R1 (*R2*), and the probability of error (*P*) were also calculated. The linearity of DMPO-AsA* was found to be better than that of DMPO-OOH from the former's higher correlation coefficient. The linearity of DMPO-AsA* in the liver tissue (*y* = −40.7*x* + 113.8, *x* = log(day), *R*1 = −0.779, (*P* < .001)) and brain (*y* = −  45.3*x* + 110.1, *x* = log(day), *R*1 = − 0.739, *P* < .001) was meaningful. 

## 4. Discussion

The significant amount of AsA is found in the body and is one of redox molecules first consumed by oxidative stress [23]. AsA is particularly an essential factor in eliminating ROS (reactive oxygen species) in which hydroxyl radicals are the most highly toxic. The lifetime of AsA* is extremely short, being measured in microseconds, which makes them extremely difficult to be detected. To date, research has been carried out via an ESR spin drum (trapping) method. However, X-band electromagnetic waves, which are emitted from whole tissues using the spin trapping method, are attenuated due to the body moisture of WIEL. Therefore, detecting the signals with this conventional method is difficult and is limited to be applied to a tissue such as brain emitting comparatively strong signals. 

In this experiment, a high purity quart cuvette was improved (modified), and by a new spin trap agent, DMPO, with a high permeability to brain tissues, the detection sensitivity of signal of AsA* in brain tissues was improved successfully by modifying the method described as follows. The permeability of X-band waves was improved by a thin tissue sample that was half of that used. WIDL was also minimized by drastically reducing the overall fluid volume (including a large quantity of moisture) added to ESR cavity for each organ from 150 *μ*L to 20–50 *μ*L. The attenuation in electromagnetic waves caused by cover glass was also reduced by making cover glass thinner (0.15 *μ*m). The ratio of the spin trap solution and the mass of each tissue slice was changed to 1.2 : 1 to improve its sensitivity. While the Masumizu method used grease on the edge of the cover glass to fix specimen, the weight of cover glass in our method was very light and its surface tension was sufficient to attach sample tissue without grease, ignoring the spectral and chemical changes induced by the grease itself. The new procedure was able to measure ESR signal from sample with high sensitivity. 

 It is demonstrated that a substantial improvement in the sensitivity of detecting DMPO-AsA* and DMPO-OOH signals occurred and that the obvious peaks of DMPO-AsA* and DMPO-OOH were detected with ESR even without oxidative stress. In a condition without oxidative stress, DMPO-OOH signals were high in heart, liver, and kidney among postmortem tissues while DMPO-AsA* signals were detected especially high in the brain and lungs samples. In the previous study [20], only the traces of AsA* adducts were detected in tissue with a low AsA concentration and it was difficult to measure them except brain. However, this study was able to show the spin adduct signals of AsA* even in tissue with a low AsA concentration without adding oxidative stress. In other words, AsA* adducts can now be detected in almost all tissue including brain, lung, heart, liver, kidney, pancreas, muscle, and skin. These results indicate that oxidative stress can be easily detected in almost all tissue as long as DMPO-AsA* adducts are used as indicators for oxidative stress. AsA* adducts are observed, when AsA reacts with hydroxyl radicals or ROS such as superoxide and also when AsA removes an electron in the regeneration process of tocopheryl radicals. On that time, these AsA* react with spin trap agents resulting in DMPO-AsA*. Reacting especially with hydroxyl radicals, AsA is known as a hydroxyl radical scavenger in the body. It is highly possible that the spin adducts of AsA* are the byproducts of these radical reactions. The peaks of DMPO-AsA* were composed of twin peaks of the same height, with *g*-values of 2.0057 and 2.0045 and a hfcc of 0.187 mT. The signals of DMPO-AsA* agree well with those values reported by Masumizu et al. [22], that is, the *g*-value and hfcc (aH) of a doublet were 2.0048 and 0.187 mT. Regarding the hydrogen radical, the *g*-values and those of its nine characteristic peaks were measured (Figures 1 and 2). Although all nine peaks were unable to be observed always, the *g*-values of peaks observed in tissue were able to be measured and compared with the standard peaks. In mouse tissue in this study, DMPO detected an AsA radicals adduct signal, superoxide adduct signal, and several other adduct signals with the same *g*-values as a hydrogen radicals adduct signal. 

ESR signals from sample tissue were identified as DMPO-OOH, DMPO-AsA*, and DMPO-H by comparing their hfcc with the reference values. Regarding DMPO-OH, a peak of equal *g*-values was detected only at trace level from tissue. Although the peak height of spin adducts in the spectrum varies depending on the concentration of spin trap agents, by thin-sliced tissue and the conditioning of spelling Q-DIP, DMPO-AsA* clearly showed better sensitivity than DMPO-OOH or DMPO-H which are detected at the same time. Since the signal of DMPO-AsA* was stable in comparison with DMPO-OOH or DMPO-H, DMPO-AsA* might be more useful in detecting intracellular oxidative stress than DMPO-OOH or DMPO-H. 

As one disadvantage, DMPO-OOH is overlapped by background signals, especially in heart and other muscles, thus making it difficult to distinguish. Conversely, DMPO-AsA* can yield clear signals, although the *g*-value overlaps that of DMPO-OOH. Therefore, DMPO is considered a useful spin trap agent, especially for detecting intracellular AsA*. The signals of DMPO-AsA* was detected in mouse brain and lung more clearly than another tissue in this ESR measurement. The signals of DMPO-OOH were detected in mouse heart and liver more clearly than other tissues. Intracellular AsA concentration in tissues (brain, lung, liver, kidney, and pancreas) is higher than extracellular AsA concentration (like in blood), because superoxide was speculated to have an extracellular source. In regards to AsA*, for DMPO-AsA*, the detection peaks for both of these were, in order from the highest to the lowest, brain > lung > liver > kidney > heart > muscle. Brain and lungs tissues have an antioxidation stress system, because both tissues are able to receive oxidation stress easily [24–27]. As for the high detection levels of AsA* in brain and lung, the concentration of AsA in organ is brain > lung > liver > kidney > heart > muscle [28]. Brain and lung are known to have an especially high AsA concentration. Therefore, there is a possibility that the peak heights of DPPMPO AsA reflect AsA concentration within respective tissue. The signal intensity ratio of DMPO-OOH was high in heart and liver tissues. 

The heights of respective peaks of DMPO-OOH at 0 days postmortem were, except blood, in order, heart (50%) and liver (50%) > brain (45%) and lungs (45%); this appeared to have matched the ranking of respective iron concentrations within each organ. The peak heights of DMPO-OOH were found to be dependent on the iron concentrations of hemoglobin being the main representative among components within each tissue. It is the experimentally found results. In a previous study, regarding iron concentrations in each organ, the blood was reported to have the highest concentrations; for a mouse of age 100 days, iron concentrations in heart, liver, kidney, and brain were 298, 254, 245, and 89 ng Fe/mg dry wt, respectively [29]. In an organism, 70% Fe exists in blood hemoglobin, while 20% to 25% exists in water-soluble ferritin and insoluble hemosiderin in the liver, spleen, bone marrow, and so forth. In blood serum, Fe exists in transferrin. Numerous reports describe multiple generation systems producing superoxide from blood. For example, a system generating superoxide is activated by phagocytes. Especially, the present study was able to continue to detect superoxide and AsA radical adduct signals in heart over 200 days postmortem (Figure 5). Further, at around 200 days, heart tissue color was found to change from black to a yellowish brown, and as an organ dries and hardens, the most of cells in the organ are presumed to die. Nevertheless, even from such tissue, the trace amounts of superoxide continued to be detected. The linearity of DMPO-AsA* was found to be better than that of DMPO-OOH from the correlation coefficients. The linearity of DMPO-AsA* liver (*y* = 40.7*x* + 113.8, *x* = log(day), *R1* = −0.779) was found to be the best (Figure 6), because it was thought that (1) the liver AsA level at death time was comparatively high and (2) the configuration of liver tissue was stable posthumously. 

As a possible system for generating superoxide over a long postmortem duration, the best candidate was thought to be the oxidation process of iron in hemoglobin. 

Hemoglobin contains four hemes; when heme iron is Fe(II), it reversibly binds with oxygen. Hemoglobin with oxygen (oxyhemoglobin) oxidizes postmortem and becomes methemoglobin containing Fe(III). At the reaction step, electrons are released and superoxide and H_2_O_2_ are generated (Haber-Weiss reaction) [30]:

(1) $$\text{Fe}\left(\text{II}\right)+{\text{O}}_{2}\to \text{Fe}\left(\text{III}\right)+{\left({{\text{O}}_{2}}^{•}\right)}^{-}$$

It is a well-known fact that hydrogen peroxide is produced from the reaction of superoxide dismutase and superoxide [31]. It has recently become clear that Fe(II) generates hydrogen peroxide (H_2_O_2_) [32, 33] due to the reaction of superoxide and H^+^(hydrogen ion) and further, that due to the reaction of (3), a hydroxyl radical is produced [34]:

(2) $$\text{Fe}\left(\text{II}\right)\;+\;{\left({{\text{O}}_{2}}^{•}\right)}^{-}\;+\;{2\text{H}}^{+}\to \text{Fe}\left(\text{III}\right)+{\text{H}}_{2}{\text{O}}_{2}$$

The iron in these reactions may be dissolved or surface bound as these reactions can occur in solution or on pyrite surface [35]. The hydrogen peroxide is generated with reaction (2) due to the Fenton reaction with Fe(II) and produces a hydroxyl radical:

(3) $$\begin{array}{cc} \text{Fe}\left(\text{II}\right)+{\text{H}}_{2}{\text{O}}_{2} & \to {{}^{•}\text{O}}_{3}\text{H}\;+\;{\text{OH}}^{-}\;+\;\text{Fe}\left(\text{III}\right) \end{array}$$

It is thought that the large amounts of generated superoxide cause further hemoglobin oxidation and that they promote further the production of methemoglobin. Ito et al., reported that iron oxidation reaction proceeds in iron protein and AsA [20]. 

 Furthermore, AsA reduces iron as the following reaction of iron-proteins and AsA; this reaction would be recycled. Possible reactions proceed in the following sequence:

(4) $$\begin{array}{c} \text{Fe}\left(\text{II}\right)\;\text{Protein}+{\text{O}}_{2}\to \;\text{Fe}\left(\text{III}\right)\;\text{Protein}+\left({\text{O}}_{2}^{•}\right) \end{array}$$

(5) $$\begin{array}{c} \begin{array}{c} \text{Fe}\left(\text{II}\right)\;\text{Protein}+{\left({{\text{O}}_{2}}^{•}\right)}^{-}+{2\text{H}}^{+}\to \text{Fe}\left(\text{III}\right)\;\text{Protein}+{\text{H}}_{2}{\text{O}}_{2} \end{array} \end{array}$$

(6) $$\begin{array}{c} \text{Fe}\left(\text{II}\right)\;\text{Protein}\;+\;{\text{H}}_{2}{\text{O}}_{2}\to {{}^{•}\text{O}}_{3}\text{H}+{\text{OH}}^{-}+\text{Fe}\left(\text{III}\right)\;\text{Protein}\\ \;\;\;\;\;\;\;+{\text{OH}}^{-}+\text{Fe}\left(\text{III}\right) \end{array}$$

(7) $$\text{Fe}\left(\text{III}\right)\;\text{Protein}+\;\text{AsA}\to \text{Fe}\left(\text{II}\right)\;\;\text{Protein}+{\text{AsA}}^{•}$$

Iron-protein recycling reaction with AsA suggested that the reactions would potentially continue for long time.

Hochstein and collaborators [36, 37] reported that the oxidation of myoglobin into ferrylmyoglobin (MbIV) is a critical event in tissue damage associated with cardiac ischaemic reperfusion states. Also, superoxide extricates free Fe from Fe-binding proteins such as ferritin, thereby assisting in the oxidation of Fe [38]. Further, in this study, DMPO-OOH originating from the blood was confirmed to be suppressed (inhibited) by the addition of citric acid, an iron chelator. It is because the content is an experimental result. In this way, in the postmortem observations in our experiments, over a long term, DMPO-OOH from tissue samples was detected, because the superoxide generation system became the main source for generating superoxide in tissue during postmortem. The linear decrease of superoxide indicated the reduction of superoxide generation in the oxidation process of Fe(II) to Fe(III). 

However, in the tissue, numerous other O_2_ generation sources were observed in addition to iron oxidation. For example, in several days postmortem, phagocytes in blood such as neutrophils, eosinophils, monocytes, and macrophages, and so forth, were thought to produce superoxide by NADPH and NADPH oxidase reactions from the stimuli of bacterial proliferation, protein degeneration, and and so forth [39]. From ESR signal of liver, the peak where the *g*-values of DMPO-OOH, DMPO-AsA*, and DMPO-H were recognized. In the liver, mitochondria in hepatocytes are active occurred, and superoxide is produced by drug metabolism [40, 41]. Superoxide apparently appears more in the liver, which is an organ most easily exposed to superoxide, because of the extremely high level of superoxide dismutase (SOD) activity reported in the liver [41]. SOD activity is also high in the liver, kidneys, and heart [42]. The main sources of superoxide in the liver are reportedly microsome P450 (P450IIE1) and NADH during the metabolism of substances like alcohol [43]. In a previous study, it was reported that the majority of superoxides originating from tissue are metabolic byproducts from mitochondria, respiration, and microsomes [40]. In the brain, superoxide is reportedly produced, when the nervous system is directly exposed to hemoglobin, which releases a large amount of iron [44]. In nerve cells, superoxide is produced by the oxidizing system of dopamine and catecholamine [45]. The generation of superoxide in the brain gives neuronal death, which is considered as a cause of damage to the nerve cells, as manifested in diseases including multiple sclerosis, the deterioration of cognitive function with aging, dementia, amyotrophic lateral sclerosis (ALS), and Alzheimer's and Parkinson's diseases [46]. 

The results of our experiments showed a tendency for increasing the peak height of DMPO-OOH, a spin adduct for superoxide, from immediately to several days after death. DMPO-OOH occurring from the superoxide production system of hepatocytes as mentioned above is also considered as the part of this increase. All mean values on three days after death were slightly above the line of the superoxide decay curve; it may be the indication of other superoxide sources than Fe. However, the marginal differences in these values imply that the postmortem occurrence of superoxide still remains a major source for generating superoxide three days after death regardless of the origin of control. For several days postmortem, cells in tissue samples remain alive and the tissues are under ischemic condition. The increase of DMPO-OOH during these postmortem days is speculated to be due to the occurrence of superoxide caused by reaction with ischemia from hypoxic condition. 

In this study, the sensitivity of detecting DMPO adduct signals in the tissue was improved using an X-band ESR and the spin trap method. For reducing the decay caused by WIDL of X-band, sample cuvette in ESR instrument was also modified and DMPO was examined as a new spin trap agent. By these improvements, spin adduct signals were detected from brain, lungs, heart, liver, kidneys, pancreas, muscles, and skin tissues and the signals were confirmed to be the genuine adduct signals of superoxide and AsA* from their *g*-values and hfcc-values of standard signal. In the postmortem follow up, DMPO-OOH, DMPO-AsA*, and DMPO-H were detected not only in the fresh tissue but also in the tissue of a mouse that had been stored more than 200 days after death in 4°C. DMPO-AsA* in liver was found to linearly decrease to logarithm of postmortem. It was related linearly to the logarithm of duration and not linearly to postmortem duration. Therefore, DMPO-AsA* signals were found to be a useful indicator estimating postmortem duration and oxidative damages in various tissue.

## References

[1] Pashinian GA, Proshut VL (1988). Determination of the time of occurrence of mechanical trauma by the EPR spectra of the bone marrow. *Sudebno-Meditsinskaia Ekspertiza*.

[2] Uzeneva RV (1989). The diagnosis of intravital mechanical trauma by the blood EPR-spectral parameters. *Sudebno-Meditsinskaya Ekspertisa*.

[3] Mil' EM, Kasparov VV, Biniukov VI, Tabatchikova NV, Borisova OA (2000). Changes in the EPR spectra of the nitrosyl complexes of blood proteins in the low-intensity whole-body irradiation of mice. *Radiatsionnaia, Biologiia, Radioecologiia*.

[4] Nakamura N, Cullings HM, Kodama Y (2006). A method to differentiate between the levels of ESR signals induced by sunlight and by ionizing radiation in teeth from atomic bomb survivors. *Radiation Research*.

[5] Quarino L, Kobilinsky L (1988). Development of a radioimmunoassay technique for the detection of human hemoglobin in dried bloodstains. *Journal of Forensic Sciences*.

[6] Türkes S, Korkmaz O, Korkmaz M (2003). Time course of the age-related alterations in stored blood. *Biophysical Chemistry*.

[7] Fujita Y, Tsuchiya K, Abe S, Takiguchi Y, Kubo SI, Sakurai H (2005). Estimation of the age of human bloodstains by electron paramagnetic resonance spectroscopy: long-term controlled experiment on the effects of environmental factors. *Forensic Science International*.

[8] Culcasi M, Pietri S, Cozzone PJ (1989). Use of 3,3,5,5-tetramethyl-1-pyrroline-1-oxide spin trap for the continuous flow ESR monitoring of hydroxyl radical generation in the ischemic and reperfused myocardium. *Biochemical and Biophysical Research Communications*.

[9] Musser SM, Fann YC, Gurbiel RJ, Hoffman BM, Chan SI (1997). Q-band electron nuclear double resonance (ENDOR) and X-band EPR of the sulfobetaine 12 heat-treated cytochrome c oxidase complex. *Journal of Biological Chemistry*.

[10] Kuppusamy P, Ohnishi ST, Numagami Y, Ohnishi T, Zweier JL (1995). Three-dimensional imaging of nitric oxide production in the rat brain subjected to ischemia-hypoxia. *Journal of Cerebral Blood Flow and Metabolism*.

[11] Yokoyama H, Lin Y, Itoh O (1999). EPR imaging for in vivo analysis of the half-life of a nitroxide radical in the hippocampus and cerebral cortex of rats after epileptic seizures. *Free Radical Biology and Medicine*.

[12] Togashi H, Matsuo T, Shinzawa H (2000). Ex vivo measurement of tissue distribution of a nitroxide radical after intravenous injection and its in vivo imaging using a rapid scan ESR-CT system. *Magnetic Resonance Imaging*.

[13] Hirayama A, Nagase S, Ueda A (2005). In vivo imaging of oxidative stress in ischemia-reperfusion renal injury using electron paramagnetic resonance. *American Journal of Physiology—Renal Physiology*.

[14] Matsumoto K, Kawai S, Chignell CF, Utsumi H (2006). Location of anthralin radical generation in mouse skin by UV-A irradiation: an estimation using microscopic EPR spectral-spatial imaging. *Magnetic Resonance in Medicine*.

[15] Takeshita K, Utsumi H, Hamada A (1991). ESR measurement of radical clearance in lung of whole mouse. *Biochemical and Biophysical Research Communications*.

[16] Utsumi H, Ichikawa K, Takeshita K (1996). In vivo ESR measurements of free radical reactions in living mice. *Journal of Toxicological Sciences*.

[17] Bielski J, Sawińska A, Pianowska J (1982). Disorders of the bioelectric activity of the brain in workers exposed to the electromagnetic fields of different frequency. *Polski Tygodnik Lekarski*.

[18] Wang X, Liu J, Yokoi I, Kohno M, Mori A (1992). Direct detection of circulating free radicals in the rat using electron spin resonance spectrometry. *Free Radical Biology and Medicine*.

[19] Ito S, Itoga K, Yamato M, Akamatsu H, Okano T (2010). The co-application effects of fullerene and ascorbic acid on UV-B irradiated mouse skin. *Toxicology*.

[20] Ito S, Mori T, Kanazawa H, Sawaguchi T (2007). Differential effects of the ascorbyl and tocopheryl derivative on the methamphetamine-induced toxic behavior and toxicity. *Toxicology*.

[21] Chi C, Tanaka R, Okuda Y (2005). Quantitative measurements of oxidative stress in mouse skin induced by X-ray irradiation. *Chemical and Pharmaceutical Bulletin*.

[22] Masumizu T, Noda Y, Mori A, Packer L (2005). Electron spin resonance assay of ascorbyl radical generation in mouse hippocampal slices during and after kainate-induced seizures. *Brain Research Protocols*.

[23] Frei B, Stocker R, Ames BN (1988). Antioxidant defenses and lipid peroxidation in human blood plasma. *Proceedings of the National Academy of Sciences of the United States of America*.

[24] Hunninghake GW, Crystal RG (1983). Cigarette smoking and lung destruction. Accumulation of neutrophils in the lungs of cigarette smokers. *American Review of Respiratory Disease*.

[25] Hogg JC (1987). Neutrophil kinetics and lung injury. *Physiological Reviews*.

[26] Olanow CW (1990). Oxidation reactions in Parkinson’s disease. *Neurology*.

[27] Cross CE, van der Vliet A, O’Neill CA, Eiserich JP (1994). Reactive oxygen species and the lung. *Lancet*.

[28] Tanaka K, Hashimoto T, Tokumaru S, Iguchi H, Kojo S (1997). Interactions between vitamin C and vitamin E are observed in tissues of inherently scorbutic rats. *Journal of Nutrition*.

[29] Massie HR, Aiello VR, Banziger V (1983). Iron accumulation and lipid peroxidation in aging C57BL/6J mice. *Experimental Gerontology*.

[30] Cohn CA, Laffers R, Schoonen MA (2006). Using yeast RNA as a probe for generation of hydroxyl radicals by earth materials. *Environmental Science and Technology*.

[31] Babior BM (2000). Phagocytes and oxidative stress. *American Journal of Medicine*.

[32] Borda MJ, Elsetinow AR, Schoonen MA, Strongin DR (2001). Pyrite-induced hydrogen peroxide formation as a driving force in the evolution of photosynthetic organisms on an early earth. *Astrobiology*.

[33] Cohn CA, Pak A, Schoonen MA, Strongin DR (2005). Quantifying hydrogen peroxide in iron-containing solutions using leuco crystal violet. *Journal of Pharmacological and Toxicological Methods*.

[34] Cohn CA, Laffers R, Simon SR, O’Riordan T, Schoonen MA (2006). Role of pyrite in formation of hydroxyl radicals in coal: possible implications for human health. *Particle and Fibre Toxicology*.

[35] O'riordan T, Schoonen MA (2006). Role of pyrite in formation of hydroxyl radicals in coal: possible implications for human health. *Particle and Fibre Toxicology*.

[36] Arduini A, Eddy L, Hochstein P (1990). The reduction of ferryl myoglobin by ergothioneine: a novel function for ergothioneine. *Archives of Biochemistry and Biophysics*.

[37] Galaris D, Eddy L, Arduini A, Cadenas E, Hochstein P (1989). Mechanisms of reoxygenation injury in myocardial infarction: implications of a myoglobin redox cycle. *Biochemical and Biophysical Research Communications*.

[38] Biemond P, Swaak AJ, van Eijk HG, Koster JF (1988). Superoxide dependent iron release from ferritin in inflammatory diseases. *Free Radical Biology and Medicine*.

[39] McCord JM (2000). The evolution of free radicals and oxidative stress. *American Journal of Medicine*.

[40] Chance B, Sies H, Boveris A (1979). Hydroperoxide metabolism in mammalian organs. *Physiological Reviews*.

[41] Cleeter MWJ, Cooper JM, Schapira AH (1992). Irreversible inhibition of mitochondrial complex I by 1-methyl-4-phenylpyridinium: evidence for free radical involvement. *Journal of Neurochemistry*.

[42] Phillips M, Camakaris J, Danks DM (1986). Comparisons of copper deficiency states in the murine mutants blotchy and brindled. Changes in copper-dependent enzyme activity in 13-day-old mice. *Biochemical Journal*.

[43] Cristiano F, de Haan JB, Iannello RC, Kola I (1995). Changes in the levels of enzymes which modulate the antioxidant balance occur during aging and correlate with cellular damage. *Mechanisms of Ageing and Development*.

[44] Braughler JM, Hall ED (1989). Central nervous system trauma and stroke. I. Biochemical considerations for oxygen radical formation and lipid peroxidation. *Free Radical Biology and Medicine*.

[45] Graham DG (1979). On the origin and significance of neuromelanin. *Archives of Pathology and Laboratory Medicine*.

[46] Keller JN, Mattson MP (1998). Roles of lipid peroxidation in modulation of cellular signaling pathways, cell dysfunction, and death in the nervous system. *Reviews in the Neurosciences*.

